# Intervenable factors associated with suicide risk in transgender persons: a respondent driven sampling study in Ontario, Canada

**DOI:** 10.1186/s12889-015-1867-2

**Published:** 2015-06-02

**Authors:** Greta R. Bauer, Ayden I. Scheim, Jake Pyne, Robb Travers, Rebecca Hammond

**Affiliations:** Epidemiology & Biostatistics, Schulich School of Medicine & Dentistry, The University of Western Ontario, London, Canada; School of Social Work & Gender Studies and Feminist Research Program, McMaster University, Hamilton, Canada; Health Sciences Program & Department of Psychology, Wilfrid Laurier University, Waterloo, Canada; Sherbourne Health Centre, Toronto, Canada

**Keywords:** Social exclusion, Transgender, Transsexual, Suicide, Suicidal behaviour, Social determinants of health, Transphobia

## Abstract

**Background:**

Across Europe, Canada, and the United States, 22–43 % of transgender (trans) people report a history of suicide attempts. We aimed to identify intervenable factors (related to social inclusion, transphobia, or sex/gender transition) associated with reduced risk of past-year suicide ideation or attempt, and to quantify the potential population health impact.

**Methods:**

The Trans PULSE respondent-driven sampling (RDS) survey collected data from trans people age 16+ in Ontario, Canada, including 380 who reported on suicide outcomes. Descriptive statistics and multivariable logistic regression models were weighted using RDS II methods. Counterfactual risk ratios and population attributable risks were estimated using model-standardized risks.

**Results:**

Among trans Ontarians, 35.1 % (95 % CI: 27.6, 42.5) seriously considered, and 11.2 % (95 % CI: 6.0, 16.4) attempted, suicide in the past year. Social support, reduced transphobia, and having any personal identification documents changed to an appropriate sex designation were associated with large relative and absolute reductions in suicide risk, as was completing a medical transition through hormones and/or surgeries (when needed). Parental support for gender identity was associated with reduced ideation. Lower self-reported transphobia (10^th^ versus 90^th^ percentile) was associated with a 66 % reduction in ideation (RR = 0.34, 95 % CI: 0.17, 0.67), and an additional 76 % reduction in attempts among those with ideation (RR = 0.24; 95 % CI: 0.07, 0.82). This corresponds to potential prevention of 160 ideations per 1000 trans persons, and 200 attempts per 1,000 with ideation, based on a hypothetical reduction of transphobia from current levels to the 10^th^ percentile.

**Conclusions:**

Large effect sizes were observed for this controlled analysis of intervenable factors, suggesting that interventions to increase social inclusion and access to medical transition, and to reduce transphobia, have the potential to contribute to substantial reductions in the extremely high prevalences of suicide ideation and attempts within trans populations. Such interventions at the population level may require policy change.

## Background

Trans (transgender or transsexual) people, who may represent up to 0.5 % of the adult population [1], have an extremely high prevalence of suicide ideation and attempts. Studies in Canada, Europe, and the United States have reported suicide attempt prevalences within the trans population that range from 22 to 43 % over the lifetime and 9 to 10 % for the past year [2–7]. In contrast, 3.7 % of all Canadians had seriously considered [8], and 0.6 % attempted [9], suicide in the past year. While completed suicide rates among trans people are unknown [10], a history of attempted suicide is the strongest predictor of completed suicide across multiple populations [11]. Demographic factors predictive of suicide attempts in the Canadian population overall include female sex, youth, chronic illness, lack of religiosity, and being unmarried [9]. These predictors may not hold within trans populations; for instance, among trans Ontarians, ideation and attempts did not differ by gender identity [2]. Moreover, while identification of demographic risk factors is helpful for targeting interventions, they are largely non-modifiable. There is an urgent need to identify intervenable risk and protective factors [12].

To date, most trans suicide research has been descriptive, or has assessed predictors of any lifetime suicide attempts [6, 7, 13]. This limits applicability of results to suicide prevention, because predictors of lifetime versus recent suicide risk, and of ideation versus attempts, may differ. Other studies have focused on patients seeking care for medical transition [14, 15], who are not representative of the entire trans population [16]. Recent longitudinal studies demonstrate reductions in psychological distress following medical transition [17, 18]. Therefore, barriers to accessing transition-related care [19] may contribute to increased suicide ideation and attempts. However, among those who have had hormonal treatment and/or sex reassignment surgery, suicide attempts and deaths remain elevated relative to the broader population [15, 20]. Thus, it appears that factors other than gender dysphoria (distress or discomfort with one’s natal sex) contribute to increased suicide risk in trans populations.

While not all trans people experience profound distress regarding their embodiment, they nearly universally report some degree of social exclusion and transphobia [21]. Trans people are subjected to invisibility in institutional settings [22], high levels of discrimination and rejection [21, 23], harassment and violence [24–26], and poverty [7, 27]. Trans-related social exclusion has been associated with increased lifetime and past-year suicide attempts [2, 6, 28], while social and family support appear to be protective [2, 29]. Determinants of suicide risk in the broader population, including depression, substance misuse, and poverty [9, 10] are also elevated among trans persons [6, 30, 31], but are consistently attributed to social exclusion and victimization [23, 25, 28, 32, 33]. Social exclusion, victimization, and trauma have been identified as key contributors to suicide disparities across marginalized populations, including sexual minorities [34–38] and Indigenous peoples [38–41].

The current study sought to identify intervenable social factors associated with suicide risk reduction for trans people.

### Theoretical model

**Fig. 1:**
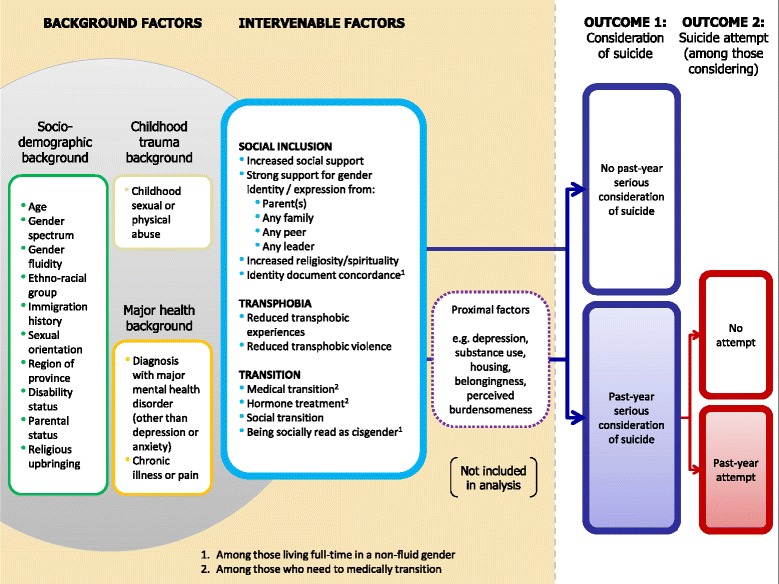
Conceptual model of intervenable social inclusion, transphobia and transition-related factors for suicide prevention among transgender people in Ontario, Canada

We present a conceptual model (Fig. 1) for two outcomes: past-year serious consideration of suicide, and – among those considering – past-year attempt. This approach reflected the possibility that factors impacting ideation and attempts may differ, as well as the pragmatic desire to both prevent suicidal ideation and to inform crisis interventions among those who are suicidal.

Variables were included as socio-demographic or background factors for the following reasons: 1) they are not amenable to change through intervention in our study population, either because they are unchangeable or because they occurred prior to age 16; 2) they are unlikely to be a result of the intervenable factors in the model, and 3) they are known or hypothesized to be associated with suicide ideation or attempts. Background factors must be controlled for as confounders in order to explore the impact of intervenable factors, so that differences observed for other factors are not based simply on differences in socio-demographic composition, illness, or history of childhood abuse.

Variables representing modifiable factors that may be targeted by potential interventions for suicide prevention within trans populations fell within three major constructs: social inclusion, transphobia (including enacted and internalized stigma, as well as violent victimization), and sex/gender transition. Their roles reflect minority stress [42], as they represent discrimination or acceptance, inclusion or exclusion, and barriers to full social participation. Moreover, all thirteen intervenable factors are social or medical determinants of health that are potentially intervenable through policy, social and/or medical intervention, but suggest different strategies. Thus, they are considered individually in this analysis rather than as overall constructs. For example, within the construct of social inclusion, interventions designed to increase parental support for gender identity or expression (e.g., family therapy, social media campaigns) would differ from those to increase identity document concordance (e.g., policy change on requirements for sex designation changes (or removal of sex designations altogether) at the federal and state/provincial levels).

Interventions on these factors have the potential to impact suicide ideation and attempts through multiple pathways. Suicide researchers have proposed models of suicidal behaviour, which focus on proximal determinants of ideation and attempts. Prominent among them is Joiner’s Interpersonal Theory of Suicide [43, 44], which accounts for a wide range of known risk factors for suicidal behaviour via three primary constructs. Suicide ideation is attributed to thwarted belongingness (resulting from social isolation) and perceived burdensomeness (e.g., resulting from family conflict or illness), and acquired capability is required for suicidal ideation to engender suicidal intent and attempts. The capability to overcome the natural disinclination to physical pain and death is acquired through previous exposure to fear and/or pain. Distal factors are posited to increase suicide risk by contributing to these proximal constructs [44]. Such distal factors are the focus of our analysis, but we include proximal factors in Fig. 1 to explicate this conceptualization. Similarly, given the frequency of transphobic experiences [21] and their profound effects on depression [30–32], housing [45], and access to services [22], we also included most of the variables traditionally defined as psychosocial risk factors as proximal factors. Together, these may mediate the effects of transphobia and other intervenable variables on ideation and/or attempts through a multitude of possible pathways that may be explored in future research.

## Methods

### Study sample

Data were collected via respondent-driven sampling (RDS) in 2009–2010 as part of the Trans PULSE Project, a community-based research study in Ontario, Canada’s most populous province. To be eligible, participants had to live, work or receive health care in Ontario, be age 16 or over, and identify as trans, broadly defined. Participants held a range of gender identities and were not required to have begun a social or medical sex/gender transition.

**Fig. 2:**
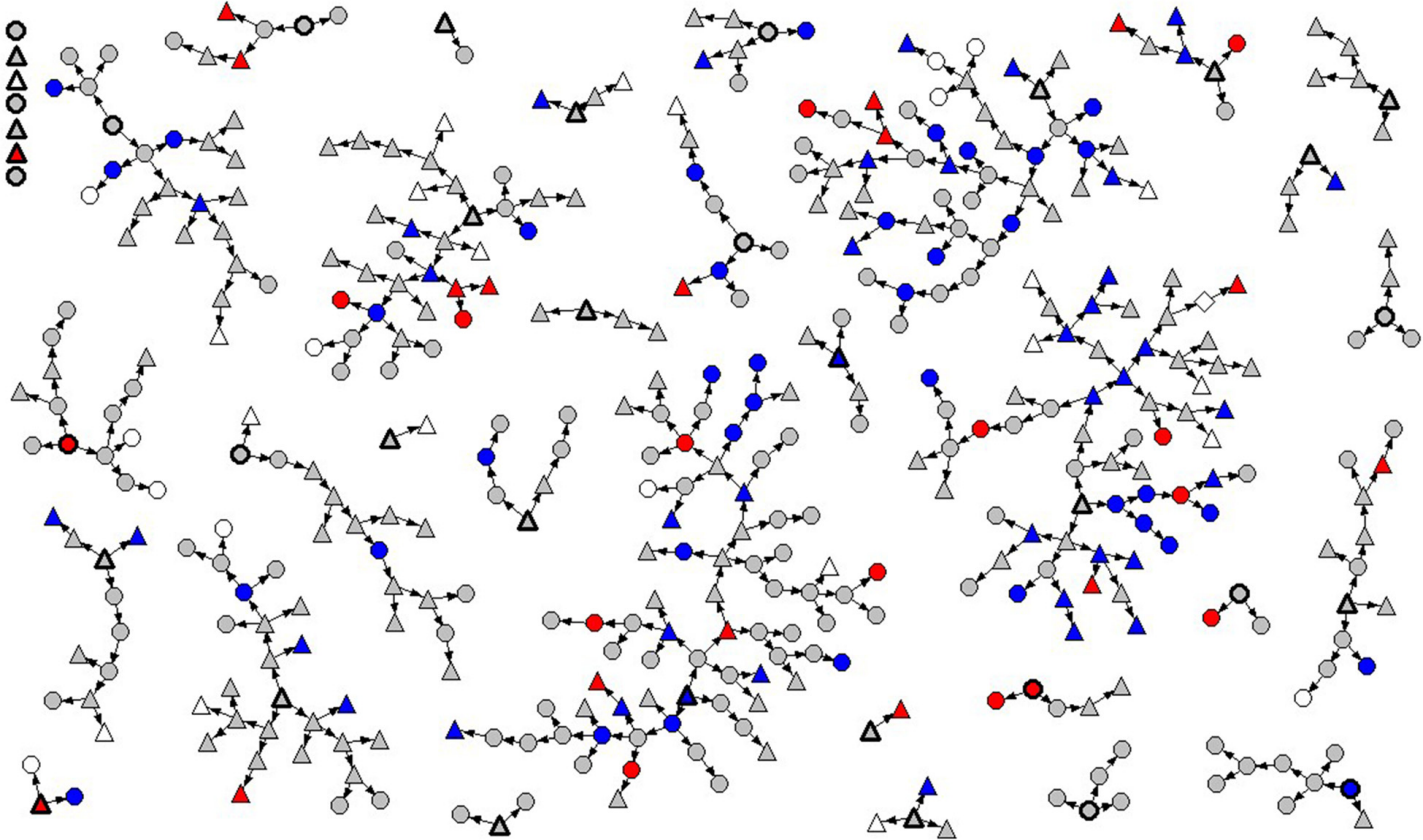
Network diagram of sample of trans people in Ontario, Canada (n = 433). Circles = male-to-female (MTF) spectrum, including genderqueer individuals. Triangles = female-to-male (FTM) spectrum, including genderqueer individuals. Grey = no past-year serious consideration of suicide. Blue = past-year serious consideration of suicide, but no attempt. Red = past-year suicide attempt(s)

RDS is a method that combines systematic chain-referral sampling with statistical analysis strategies that account for differential levels of connectedness as well as non-independence within recruitment chains. This method was chosen as trans persons in Ontario were at least moderately networked, either in person or electronically, but constituted a hidden population that could not be randomly sampled. Moreover, given the lack of data on trans people, there was community motivation to participate, including to recruit others. Sampling began with 16 initial seeds, with 22 additional seeds enrolled once it was certain that sufficient recruitment chain length could be obtained. Each participant could recruit additional participants using a tracked coupon system, wherein three coupons were issued to each participant for distribution to eligible individuals. This allowed the research team to identify the network structure (i.e., who recruited whom) while allowing participants to remain anonymous if desired. Data collection continued for one year (n = 433), with a maximum chain length of ten waves. A network diagram of the sample is presented in Fig. 2. Our analysis is based on 380 participants (87.8 %) who completed items on past-year suicide ideation and attempts. Ethics approval for the project was provided by Research Ethics Boards at The University of Western Ontario and Wilfrid Laurier University.

### Survey and measures

Measures were derived from self-report data collected through a multi-mode survey (with visually identical online or paper versions), which could be completed anonymously and typically took 60 to 90 minutes. The survey was pre-tested by 16 members of the project’s Community Engagement Team for content validity, clarity and length. A copy of the survey is available online (http://transpulseproject.ca/resources/trans-pulse-survey/).

#### Outcomes

Past-year suicidal ideation was coded from items that asked participants “Have you ever seriously considered committing suicide or taking your own life?” and “If yes, has this happened in the past 12 months?” Similar follow-up questions for those who indicated ideation asked “Have you ever attempted to commit suicide or tried taking your own life?” and “If yes, did this happen in the past 12 months?”

#### Background variables

*Age, disability status*, *parental status,* and *chronic illness/pain* were indicated by participant self-report. *Gender spectrum* was coded into two categories that each represent a spectrum of identities. Female-to-male spectrum individuals were those assigned female at birth, but who now identify as men or another non-female identity (e.g., genderqueer, bigender); similarly, male-to-female spectrum participants were those assigned male at birth who now identify as women, trans girls, two-spirit, or other non-male identities. *Gender fluidity* was coded as a separate dichotomous measure from a check-all-that-apply identity item into two categories: holding a more conventional gender identity (male or primarily masculine, female or primarily feminine), or only gender-fluid or third-gender identities. *Ethno-racial group* was comprised of three groups: participants were grouped as Aboriginal if they indicated they were First Nations, Métis, or Inuit, or another Indigenous ethnicity; non-Aboriginal participants were classified as white or racialized based on an ethno-racial background item, a write-in question describing background, and indication as to whether or not they were perceived by others as a person of colour. In Ontario, the term “racialized” is preferred over racial minority, visible minority, person of colour or non-White as it expresses race as a social construct rather than a description on perceived biological traits” [46]. *Immigration history* was approximated by self-report of whether the participant was born in Canada. *Sexual orientation* was coded as sexual minority based on either identification as gay, lesbian or bisexual/pansexual, or having a past-year sex partner of the same gender. *Region of province* was classified based on first letter of postal code. *Strong religious upbringing* was defined as responding “quite” or “extremely” to the question “How religious or faith-based was your upbringing?” *Childhood abuse* prior to age 16 was indicated in two items describing physical and sexual abuse experiences. *Major mental health disorders* were coded using a self-reported checklist based on any prior diagnosis (e.g., bipolar disorder, schizophrenia, borderline personality disorder). Depression and anxiety disorders were excluded, as they are more likely to result from intervenable factors under study, and we hypothesized they would partially mediate their effects on suicide ideation or attempt; if we were to control for these mediators we would then remove a portion of the causal effect and would produce estimates for the effects of our intervenable factors as enacted only through pathways other than depression or anxiety.

#### Intervenable variables – social inclusion

*Social support* was assessed using the 19-item Medical Outcomes Study Social Support Scale [47]. Items were averaged, with possible scale values from 1 to 5 (Cronbach α = 0.97). *Strong support for gender* was coded for participants’ parents by combining self-report of either “very strong” support for gender identity or expression experienced from a parent or parents, or (for those who were not out to their parents) self-report of expectation of such support. Other gender support variables were similarly coded. “Peers” averaged completed items on friends, co-workers, or classmates, and “Leaders” on schools, teachers, supervisors or employers. *Current religiosity or spirituality* was measured on a 6-point Likert scale ranging from “not at all” to “extremely”. *Having one or more identity documents concordant with lived gender* was coded for the participant sub-group who had socially transitioned and lived full-time in a male/masculine or female/feminine gender, based on having at least one document with a sex/gender designation (“M” or “F”) matching one’s lived gender. Identity documents included federal and provincial identification (e.g. drivers license, passport, Indian Status card, military ID).

#### Intervenable variables –transphobia

*Transphobia* was assessed using an 11-item scale [21]; it included items on enacted and internalized stigma as well as victimization, such as police harassment or feeling that being trans hurt or embarrassed one’s family. Items were summed, with possible values ranging from 0 to 33 (Cronbach α = 0.81). *Transphobic harassment and violence* was defined as self-report of physical or sexual assault for being trans (analogous to assault as a hate crime); report of verbal harassment or threats related to being trans, but not of assault; and report of none of these.

#### Intervenable variables –transition

*Medical transition status* was self-reported as having completed a medical transition (self-defined), being in the process, planning to transition but not yet having begun, and either not planning, being unsure, or indicating that the concept of “transition” did not apply. Completing a medical transition involved varying hormone and/or surgical treatments [16]. *Hormone use* was self-reported. *Social transition status* was coded as living in one’s felt gender full-time, part-time, or not at all. *Being perceived as cisgender (non-trans)* was coded for those living full-time in a non-fluid gender, based on a survey item that asked how often others knew you were trans without being told.

### Statistical analysis

Since eligible trans persons who knew fewer potential participants were less likely to be sampled than those with large network sizes, RDS II weights [48] were calculated based on the inverse of each participant’s degree (personal network size), rescaled to sum to the total sample size of 433. This approach has been shown to produce frequency estimates that are asymptotically unbiased [49]. Here weighted statistics can be interpreted to apply to the population of networked (know at least one other eligible person) trans people age 16 and over in Ontario. All analyses were weighted, and adjusted for clustering by shared recruiter to account for non-independence within recruitment chains. Weighted frequencies or means, along with 95 % confidence intervals, were calculated for all background and potential intervention variables using SAS version 9.3 [50].

Prior to regression, simple imputation was used for background variables to reduce data loss in a complete case analysis. Of 380 participants with outcome data, 30 were missing data for one background covariate and 5 for more than one.

Multivariable logistic regression models were fitted for each intervenable factor variable separately, controlling for all background variables, using SAS-callable SUDAAN version 11.0 [51]. Thus, 13 logistic regression models were fitted for the 13 intervenable factor variables for the suicidal ideation outcome, and an additional 13 models for the suicide attempt outcome; all models similarly controlled for background variables, but not for other intervenable variables, as mediated pathways between these variables are unknown. A domain analysis was used in order to limit the second analysis to the sample subgroup with suicidal ideation.

All categorical variables were dummy-coded to allow for independent estimation of effects for each group. Reference categories were chosen so that contrasts reflected effects in the direction of desired potential intervention effects (e.g., protecting from assault, increasing social support). Continuous variables were entered into the models as continuous. Since average marginal risks are estimated for specific points in the distribution (as if the entire trans population – standardized to the background variables – was at that level), the 10^th^ and 90^th^ percentiles were used as points for estimation since participant values did not necessarily cover the range of a scale and the extremes may not be achievable with any intervention (e.g., no transphobia would require not even having heard once that trans people were not normal).

Model-standardized risks and risk ratios for past-year suicidal ideation and attempts (standardized to the weighted sample or subsample on all background factors) were estimated for each of the thirteen intervenable variables under the counterfactual conditions where all participants were exposed or alternately all were unexposed to a dichotomous intervenable factor, or to an intervention target and reference level of a categorical factor. For continuous variables, these counterfactual risks were estimated for the 10^th^ and 90^th^ percentile levels. Standardized risks and risk ratios, and their 95 % confidence intervals, were calculated by Graubard and Korn’s method [52] using the ADJRR option in SUDAAN PROC RLOGIST [51]. Where effects were statistically significant for a variable, counterfactual population attributable risks (cPARs) were then calculated by taking the weighted prevalence of the outcome in the sample (factual) and subtracting the model-standardized risk (counterfactual) based on having the entire population at intervention target levels of the exposure. These represent the potential proportion of the trans population affected (e.g., outcome averted) under the intervention target condition (e.g., all have parental support for gender). Counterfactual population attributable risk proportions (c%PARs) were similarly estimated by dividing cPAR by the weighted prevalence of the outcome in the sample; these represent the proportion of cases potentially averted within the trans population. C%PARs for different variables will sum to more than 100 %, given that a case of ideation or suicide attempt could be prevented through multiple means.

## Results

Means or frequencies for sociodemographics and background covariates are presented in Table 1. Frequencies for intervenable variables and past-year suicide ideation and attempt are presented in Table 2. In the past year, 35.1 % (95 % CI: 27.6, 42.5) had seriously considered suicide, and 11.2 % (95 % CI: 6.0, 16.4) reported that they had attempted it.

**Table 1:** Weighted background characteristics of trans people in Ontario, Canada (n=380)

Background variables		
	n = 380	
	% or $\overline{x}$	95 % CI
Age, years $\left(\overline{x}\right)$	32.7	(30.5, 35.0)
Gender spectrum (%)		
Female-to-male spectrum	52.6	(44.4, 60.8)
Male-to-female spectrum	47.4	(39.2, 55.6)
Gender fluidity (%)		
Primarily fluid or third gender identity	17.7	(11.3, 24.1)
Primarily masculine or feminine identity	82.3	(75.9, 88.7)
Ethnoracial group (%)		
Aboriginal	6.6	(3.2, 9.9)
Non-Aboriginal white	77.3	(71.0, 83.6)
Non-Aboriginal racialized	16.1	(10.6, 21.7)
Place of birth (%)		
Canada	80.3	(74.1, 86.6)
Outside of Canada	19.7	(13.4, 25.9)
Sexual orientation (%)		
Lesbian, gay, bisexual or MSM/WSW	66.7	(59.2, 74.2)
Straight	33.3	(25.8, 40.8)
Region of residence (%)		
Southeastern Ontario	10.8	(5.9, 15.7)
South Central Ontario	16.4	(10.1, 22.7)
Metropolitan Toronto	38.3	(30.3, 46.3)
Southwestern Ontario	27.2	(18.7, 35.8)
Northern Ontario	7.3	(2.9, 11.6)
Visual, hearing, communication or mobility disability (%)	12.4	(8.2, 16.7)
Parental status (young or grown children) (%)	25.6	(18.2, 32.9)
Strong religious upbringing (%)	23.7	(16.8, 30.7)
Childhood physical or sexual abuse (%)	70.7	(63.5, 78.0)
Major mental health disorder (%)	19.1	(13.4, 24.7)
Living with chronic illness or chronic pain (%)	18.0	(12.1, 23.9)

**Table 2:** Means or frequencies of intervenable variables (social support, transphobia, and transition) and suicidality among trans people in Ontario, Canada (n=380)

	n = 380	
	% or $\overline{x}$	95 % CI
Social Support		
**Social support** $\left(\overline{x}\right)$	3.5	(3.3, 3.6)
Strong parental support for gender (%)^a^	28.5	(21.6, 35.4)
Strong family support for gender (%)^a^	60.5	(52.2, 68.7)
Strong support from leaders (%)^a^	47.4	(38.1, 56.8)
Strong support from peers (%)^a^	86.1	(80.3, 91.9)
Religiosity or spirituality $\left(\overline{x}\right)$	3.0	(2.7, 3.3)
Having ≥1 ID concordant with lived gender (%)^b^	51.1	(41.8, 60.5)
**Transphobia**		
Transphobia scale $\left(\overline{x}\right)$	14.1	(13.0, 15.1)
Transphobic violence (%)		
None	44.0	(35.6, 52.3)
Verbal harassment or threats	34.9	(27.5, 42.2)
Physical or sexual assault	21.2	(15.0, 27.3)
**Transition**		
Medical transition status (%)^c^		
Completed	35.3	(27.6, 42.9)
In process	31.8	(24.6, 39.0)
Planning, but not begun	32.9	(24.9, 40.9)
Social transition status (%)		
Full-time	53.3	(44.7, 61.9)
Part-time	26.9	(19.8, 34.1)
Not living in core gender	19.8	(12.1, 27.4)
Current hormone use (%)^c^	57.6	(49.1, 66.1)
Being socially seen as cisgender (%)^b^		
Almost always or always	45.5	(35.3, 55.6)
About half time or often	25.5	(17.7, 33.2)
Rarely or never	29.0	(19.7, 38.4)
**Suicidality**		
Seriously considered suicide, past yr (%)	35.1	(27.6, 42.5)
Attempted suicide, past yr (%)	11.2	(6.0, 16.4)

^a^Support variables include either indication of – or expectation of – strong support

^b^among those living full-time in a non-fluid gender (n = 251)

^c^among those reporting need to medically transition sex (n = 346)

Findings on model-standardized risks are presented in Table 3 for suicide ideation and in Table 4 for suicide attempts (among those with ideation), along with model-standardized relative risks and population attributable risks. Average relative effects on individuals, and at a trans population level, were sometimes strong. High levels of social support (90th percentile) versus low levels (10^th^ percentile) were significantly associated with a 49 % reduction in suicide ideation (RR = 0.51; 95 % CI: 0.28, 0.94), and with a further 82 % reduction in attempt risk among those with ideation (RR = 0.18; 95 % CI: 0.04, 0.73). This would be associated with potential prevention of 100 cases of ideation per 1,000 trans persons (cPAR = 0.10), and a further prevention of 220 attempts per 1,000 trans persons considering suicide (cPAR = 0.22). Among sources of strong support for gender, only support from parents was statistically significantly associated with reduced risk, with RR = 0.43 (95 % CI: 0.26, 0.73) for past-year ideation, and no additional statistically significant effect on the risk of attempts among those with ideation. At a population level, this corresponds to potential prevention of 170 trans persons per 1,000 (cPAR = 0.17) from seriously considering suicide (and thus also reducing the risk of attempt through lowering the proportion at risk). Interestingly, strong support from leaders such as supervisors or teachers was significantly associated with an increased risk of attempts among those with ideation (RR = 5.24; 95 % CI: 2.20, 12.46). Having one or more identity documents concordant with lived gender was significantly associated with reductions in past-year ideation (RR = 0.56; 95 % CI: 0.35, 0.90) and attempts (RR = 0.26, 95 % CI: 0.11, 0.62), with the potential to prevent 90 cases of ideation per 1,000 trans persons (cPAR = 0.09), and 230 attempts per 1,000 with ideation (cPAR = 0.23). Religiosity was not associated with suicidality.

**Table 3:** Model-standardized risks for intervenable variables on suicidal ideation among trans people in Ontario, Canada (n = 380)

Potential intervention factor	Number	R_F_^a^	Model-standardized RR^d^		Counterfactual population attributable risk (R_F_ - R_1_)^f^	Counterfactual population attributable risk proportion $\frac{{\left({\mathrm{R}}_{\mathrm{F}}-{\mathrm{R}}_1\right)}^{\mathrm{g}}}{{\mathrm{R}}_{\mathrm{F}}}$
		R_1_^b^				
		R_0_^c^	RR	95 % CI^e^		
Social inclusion						
**Social support**	377	0.34				
90th percentile (4.895)		0.24	0.51	(0.28, 0.94)	0.10	29 %
10th percentile (1.947)		0.47	1.00	--		
Any strong parental support for gender^h^	324	0.36				
Yes		0.19	0.43	(0.26, 0.73)	0.17	47 %
No		0.43	1.00	--		
Any strong family support for gender^h^	368	0.35				
Yes		0.29	0.66	(0.43, 1.00)		
No		0.44	1.00	--		
Any strong support from leaders^h^	259	0.32				
Yes		0.32	1.02	(0.62, 1.48)		
No		0.32	1.00	--		
Any strong support from peers^h^	366	0.35				
Yes		0.33	0.67	(0.40, 1.11)		
No		0.49	1.00	--		
Religiosity or spirituality	376	0.35				
90th percentile (5)		0.29	0.69	(0.42, 1.15)		
10th percentile (1)		0.41	1.00	--		
Having ≥1 ID concordant with lived gender^i^	254	0.33				
Yes		0.24	0.56	(0.35, 0.90)	0.09	27 %
No		0.43	1.00	--		
**Transphobia**						
Transphobia scale	374	0.34				
10th percentile (5)		0.18	0.34	(0.17 0.67)	0.16	47 %
90th percentile (23)		0.52	1.00	--		
Transphobic harassment and violence	380	0.35				
None		0.34	0.68	(0.41, 1.13)	0.01	3 %
Verbal harassment or threats		0.27	0.54	(0.35, 0.85)		
Physical or sexual assault		0.50	1.00	--		
**Transition**						
Medical transition status^j^	346	0.39				
Completed		0.21	0.38	(0.22, 0.66)	0.17	44 %
In process		0.40	0.71	(0.48, 1.05)		
Planning, but not begun		0.56	1.00			
Current hormone use^j^	306	0.38	0.52	(0.37, 0.75)	0.10	26 %
Yes		0.28				
No		0.53	1.00	--		
Social transition status	378	0.35				
Full-time		0.32	0.64	(0.38, 1.07)		
Part-time		0.33	0.68	(0.40, 1.15)		
Not living in core gender		0.49	1.00	--		
Being socially seen as cisgender^i^	223	0.34				
Almost always or always		0.26	0.73	(0.37, 1.47)		
About half time or often		0.46	1.29	(0.74, 2.26)		
Rarely or never		0.36	1.00	--		

Risks are standardized to sociodemographic factors, childhood trauma factors and major health-related background factors. These included: age, gender spectrum, gender fluidity, ethno-racial group, immigration history, sexual orientation, region of province, disability status, parental status, religious upbringing, childhood sexual or physical abuse, diagnosis with major mental health disorder (excluding depression or anxiety), and chronic illness or pain

^a^R_F_ = estimated risk of past-year suicidal ideation in the factual trans population of Ontario

^b^R_1_ = model-standardized estimated risk of past-year suicidal ideation in the trans population of Ontario, under the same distribution of background factors, but where all members have a positive value of the potential intervention (e.g., high support, no transphobic violence)

^c^R_0_ = model-standardized estimated risk of past-year suicidal ideation in the trans population of Ontario, under the same distribution of background factors, but where all members have a negative value of the potential intervention (e.g., low support, exposure to transphobic violence)

^d^Will vary slightly from R_1_/R_0_ as are calculated as average of individual marginal risk ratios, rather than the ratio of model-standardized risks

^e^95 % confidence intervals from Taylor series linearization methods in SUDAAN

^f^Counterfactual population attributable risk = potential proportion of population protected from suicidal ideation by a hypothetical move from population levels of this factor to an intervention level

^g^Counterfactual population attributable risk proportion = potential proportion of outcomes that could be averted under a change in exposure frequency

^h^Support variables include either indication of – or expectation of – strong support

^i^among those living full-time in a non-fluid gender (n = 251)

^j^among those reporting need to medically transition sex (n = 346)

**Table 4:** Model-standardized risks for intervenable variables on suicide attempts among trans people who have seriously considered suicide, Ontario, Canada (n = 110)

Potential intervention factor	number	R_F_^a^	Model-standardized RR^d^		Counterfactual population attributable risk (R_F_ - R_1_)^f^	Counterfactual population attributable risk proportion $\frac{{\left({\mathrm{R}}_{\mathrm{F}}-{\mathrm{R}}_1\right)}^{\mathrm{g}}}{{\mathrm{R}}_{\mathrm{F}}}$
		R_1_^b^				
		R_0_^c^	RR	95 % CI^e^		
Social inclusion						
**Social support**	109	0.30				
90th percentile (4.895)		0.08	0.18	(0.04, 0.73)	0.22	73 %
10th percentile (1.947)		0.47	1.00	--		
Any strong parental support for gender^h^	99	0.31				
Yes		0.10	0.29	(0.07, 1.24)		
No		0.35	1.00	--		
Any strong family support for gender^h^	106	0.30				
Yes		0.27	0.81	(0.41, 1.62)		
No		0.33	1.00	--		
Any strong support from leaders^h^	72	0.34				
Yes		0.58	5.24	(2.20, 12.46)	−0.24	n/a
No		0.11	1.00	--		
Any strong support from peers^h^	106	0.30				
Yes		0.32	1.34	(0.43, 4.23)		
No		0.24	1.00	--		
Religiosity or spirituality	109	0.30				
90th percentile (5)		0.20	0.52	(0.12, 2.18)		
10th percentile (1)		0.39	1.00	--		
Having ≥1 ID concordant with lived gender^i^	55	0.37				
Yes		0.14	0.26	(0.11, 0.62)	0.23	62 %
No		0.54	1.00	--		
**Transphobia**						
Transphobia scale	108	0.31				
10th percentile (5)		0.11	0.24	(0.07, 0.82)	0.20	65 %
90th percentile (23)		0.45	1.00	--		
Transphobic harassment and violence	110	0.32				
None		0.18	0.30	(0.08, 1.16)	0.14	44 %
Verbal harassment or threats		0.18	0.31	(0.11, 0.83)		
Physical or sexual assault		0.59	1.00	--		
**Transition**						
Medical transition status^j^	100	0.35				
Completed		0.11	0.51	(0.07, 3.74)	0.24	69 %
In process		0.65	2.91	(1.47, 5.76)		
Planning, but not begun		0.22	1.00	--		
Current hormone use^j^	98	0.36				
Yes		0.30	0.76	(0.41, 1.39)		
No		0.40	1.00	--		
Social transition status	109	0.31				
Full-time		0.45	5.30	(0.66, 42.68)		
Part-time		0.21	2.53	(0.34, 18.60)		
Not living in core gender		0.08	1.00	--		
Being socially seen as cisgender^i^	56	0.36				
Almost always or always		0.48	0.98	(0.58, 1.64)		
About half time or often		0.14	0.28	(0.02, 3.26)		
Rarely or never		0.49	1.00	--		

Risks are standardized to sociodemographic factors, childhood trauma factors and major health-related background factors. These included: age, gender spectrum, gender fluidity, ethno-racial group, immigration history, sexual orientation, region of province, disability status, parental status, religious upbringing, childhood sexual or physical abuse, diagnosis with major mental health disorder (excluding depression or anxiety), and chronic illness or pain

^a^R_F_ = estimated risk of past-year suicidal ideation in the factual trans population of Ontario

^b^R_1_ = model-standardized estimated risk of past-year suicidal ideation in the trans population of Ontario, under the same distribution of background factors, but where all members have a positive value of the potential intervention (e.g., high support, no transphobic violence)

^c^R_0_ = model-standardized estimated risk of past-year suicidal ideation in the trans population of Ontario, under the same distribution of background factors, but where all members have a negative value of the potential intervention (e.g., low support, exposure to transphobic violence)

^d^Will vary slightly from R_1_/R_0_ as are calculated as average of individual marginal risk ratios, rather than the ratio of model-standardized risks

^e^95 % confidence intervals from Taylor series linearization methods in SUDAAN

^f^Counterfactual population attributable risk = potential proportion of population protected from suicidal ideation by a hypothetical move from population levels of this factor to an intervention level

^g^Counterfactual population attributable risk proportion = potential proportion of outcomes that could be averted under a change in exposure frequency

^h^Support variables include either indication of – or expectation of – strong support

^i^among those living full-time in a non-fluid gender (n = 57)

^j^among those reporting need to medically transition sex (n = 100)

Both transphobia variables in the analysis were associated with ideation and attempts, with lower transphobia associated with reduced risk. Lower overall transphobia (10^th^ percentile vs. 90^th^) was statistically significantly associated with a 66 % relative risk reduction of past-year ideation (RR = 0.34; 95 % CI: 0.17, 0.67) and an additional 76 % relative risk reduction (RR = 0.24; 95 % CI: 0.07, 0.82) for attempts. This represents a potential prevention of 160 cases of ideation per 1,000 trans persons (cPAR = 0.16), and potential prevention of 200 attempts per 1,000 with ideation (cPAR = 0.20).

Medical transition variables, but not social transition or being perceived as cisgender, were associated with suicidality. Among those who desired medical transition, those on hormone therapy were about half as likely to have seriously considered suicide (RR = 0.52; 95 % CI: 0.37, 0.75). The process of medically transitioning overall was more complex, with a monotonic reduction in suicide ideation from planning to transition vs. being in process, vs. completing. However, among the sub-group with ideation, being in the process of transitioning was significantly associated with increased risk of an attempt (RR = 2.91; 95 % CI: 1.48, 5.76) in comparison with those who were planning to transition but had not yet begun. We did not observe an increased risk in this sub-group among those who completed a medical transition (RR = 0.51; 0.07, 3.74). Completing a medical transition had beneficial individual and population effects. It was associated with a 62 % relative risk reduction (RR = 0.38; 95 % CI: 0.22, 0.66) in ideation. On a trans population level, to facilitate completion of medical transition (when desired) would correspond to preventing 170 cases of ideation per year per 1,000 trans persons (cPAR = 0.17), representing 44 % of ideation (c%PAR = 0.44), and further preventing 240 attempts per 1,000 with ideation (cPAR = 0.24) or 69 % of attempts in this group (c%PAR = 0.69).

## Discussion

Our findings provide evidence that social inclusion (social support, gender-specific support from parents, identity documents), protection from transphobia (interpersonal, violence), and undergoing medical transition have the potential for sizeable effects on the high rates of suicide ideation and attempts in trans communities. In contrast, we did not find statistically significant effects for social transition, gender support from sources other than parents, or religiosity/spirituality, other than an unexpected finding regarding strong gender support from leaders. Given that statistical power was not high, as evidenced by the width of our confidence intervals, a lack of statistical significance does not mean that these other factors should be dismissed, as smaller effects may exist below the threshold for detection.

Our results provide support for the potentially strong impact of trans-specific discrimination or harassment (e.g., experiences of transphobia), interpersonal factors (e.g., strong parental support for gender identity or expression) and structural factors (e.g., having an identity document with a gender marker concordant with one’s lived gender) on suicide ideation or attempts. This reinforces our earlier descriptive findings that risk of suicide ideation and attempts varied greatly among trans people [2], and reinforces the need to look beyond proximal determinants toward sites of early prevention or intervention. It is not clear to what extent results from this study may also apply to gender non-conforming cisgender persons, but we note that among sexual minority youth, early gender non-conformity has been associated with increased suicidal behaviour or risk, a process that may be mediated by gender harassment or bullying [53–55], or by parental disapproval of gender expression [55].

The large effect sizes observed support the possibility for preventing suicidal ideation and attempts in a large number of individuals. Using the transphobia results as an example, combining the population effects of a reduction in ideation and a reduction in attempt risk among the reduced cases of ideation, and given a population estimate of 53,500 trans adults in Ontario [26], we would estimate that reducing experiences of transphobia could prevent 8,560 trans persons in the province from experiencing suicidal ideation and 4,601 persons from a suicide attempt within a year.

Our results represent the most detailed analysis of this issue to date; our study was based on a respondent-driven sample of trans people from a large provincial geographic area. The analysis takes account of differential probability of recruitment related to differences in network size, but other biases unrelated to network size may remain [56]. Our use of past-year suicide-related measures represents an improvement over studies that used lifetime measures, as we are able to analyse impact on recent or current risk, which is most relevant to prevention. However, temporality remains a concern. It is possible that some potential causes occurring in the past year followed rather than preceded the outcome. This is one potential explanation of the unusual finding of support from a leader (teacher, supervisor, institution) being associated with increased suicide attempts among those with ideation, in that an attempt may trigger the involvement of leaders. Moreover, as we were unable to determine the exact sequences of events for each participant, it is likely that we have partially controlled for some mediating effects or not controlled for some confounding. For example, borderline personality disorder is adjusted for as a confounder, though it is possible that for some participants its etiology includes experiences of transphobia such as those we assess, and it may thus play a mediating role. Despite these limitations, we attempted to address temporality within a cross-sectional design through time designations within questionnaire items (e.g., childhood abuse prior to the age for inclusion in this study) and use of past-year outcomes.

Our finding that completing a medical transition was associated with reduced risk has implications for interpretation of existing studies on completed suicides. Because trans people are not identifiable in death records, and because completed suicides may occur among those who know they are trans but are not known by family members to be trans, valid studies of completed suicides have only been done where patient records from gender clinics have been matched to population death records (e.g., in Sweden [15]). Our results suggest these estimates of completed suicide among those who have medically transitioned likely underestimate the risk of suicide among broader trans communities.

As all surveys are, by definition, studies of survivors, survival bias remains an issue. Frequencies for attempts will likely be underestimated. Factors that predict lethality may be missed, if those who completed suicide differ from those who survived attempts. Given that we assessed suicide attempts only among those who indicated past-year serious consideration, our data may also have missed additional attempts that were impulsive and unplanned. Moreover, past-year prevalence may not represent a first incident of suicide ideation or attempt; thus, this analysis cannot distinguish between factors that lead one to first become suicidal versus to continue being suicidal.

Proximal factors theorized and demonstrated to increase risk of suicide ideation and/or attempts (e.g., risk factors from epidemiological research and interpersonal factors from Joiner’s Interpersonal Model [43]) were conceptualized as mediators, but not included in these analyses. Moreover, our analysis could not disaggregate effects of intervenable variables on other intervenable variables. For example, it is possible that increased parental support for gender may affect whether or not someone is able to medically transition; it is also possible that medical transition results in increased parental support for gender, as parents are able to more clearly see their child in their felt gender. These areas represent opportunities for future research. In general, suicide research regarding gender and sexual minorities has tended to overlook existing theoretical frameworks within suicidology [57], though they are not incompatible with other frameworks or methods. Future research with trans populations could draw on interdisciplinary theories as well as evidence from trans-specific and broader population research on suicide ideation and attempts to study mediated pathways.

Future prospective cohort studies of broadly defined trans populations are needed to alleviate many of the limitations of this and other studies. With prospective data, we may be able to differentiate between factors that cause initial ideation and factors that prolong its duration, as well as those that lead to first and repeated attempts. We could also begin to study completed suicides in a non-clinical trans population, at least among those who are willing to identify as trans to researchers. Moreover, with clear information on temporality, we would be able to design better controlled and more valid analyses, and to examine mediated pathways (including pathways between intervenable factors as well as proximal factors) to better understand the process through which social marginalization may impact suicide ideation and attempt.

Our goal of evaluating intervenable factors should be interpreted as a screening of potential strategies rather than an analysis of actual population intervention effects. While background factors are structured to represent those that are in the past, unchangeable, or not likely to change in response to other factors in the model, our analytic approach then considers intervenable factors singly; it was not possible to tease apart causal pathways among these factors and combining them into one model would by default serve to prioritize the proximal causal factors while reducing the effect sizes of other potentially important causes [58] (a general effect of multivariable models that is not often explicated but is commonly understood with regard to control for a mediator reducing a causal effect [59, 60]). Depending upon inter-individual variation, as in all individual-level studies, also results in an inability to detect simultaneous effects at the group level [61]. For example, reducing transphobic assaults from the current prevalence of 21.2 to 0 % may affect suicide risk not only by saving individuals from the trauma of hate-based assault, but may have additional effects on these individuals and others based on living in a society where transphobic assaults do not occur, versus where they are common.

## Conclusions

Our findings support a strong effect for social exclusion, discrimination and lack of medical transition (for those needing it) on suicide ideation and attempts, and potentially on the survival of trans persons. This adds support to the larger discussion regarding social impacts on suicide risk in groups experiencing marginalization, such as Indigenous communities and sexual minorities. Our team has previously published recommendations for suicide prevention efforts with trans persons, based on descriptive analyses of these data [2]. The present analysis provides stronger support for those recommendations, including attention to social support and protection from discrimination, by showing that these effects remain after adjusting for potential confounding by background. It also suggests additional targets for intervention. Specifically, while gender recognition is recognized as a human right for trans people in Ontario [62], we have provided the first evidence of its potential to reduce suicidality. Since our data were collected, the surgical requirement for changing the sex/gender designation on an Ontario birth certificate has been eliminated. Such legal and policy changes can be considered public health interventions worthy of longer-term evaluation. In addition, parental support has been previously associated with reduced suicide risk for sexual minority [63] and trans [64] youth, but our results demonstrate the importance of parental support for gender identity among adults, suggesting a need for all-ages family interventions. Finally, we found that among those reporting a need to medically transition through hormones and/or surgeries, suicidality was substantially reduced among those who had completed a medical transition (this involved varying procedures based on personal needs [16]). Despite potentially large reductions in risk for those completing medical transition, the period of being in process did not represent a clear mid-point in risk. While suicidal ideation was significantly reduced for those in process versus those who were planning to transition but had not begun, among the sub-group considering suicide the attempt rate was highest among those in process. These results call into question the safety of clinical practices that delay transition treatments until depressive symptoms or suicidality are well-controlled, and of procedural practices that require or result in long delays in the medical transition process, but also suggest need for supports for those who may feel suicidal while in the process of transitioning.

Our findings strongly suggest that interventions aimed at increasing social inclusion, reducing transphobic discrimination and violence, and facilitating access to medical transition should be considered as part of a comprehensive approach to suicide prevention in trans populations, and evaluated to assess effectiveness. Such interventions need not supplant individual-level or therapeutic approaches (e.g., psychotherapy, crisis services), but have the potential to reduce suicide ideation and attempts by targeting stigma and social exclusion as fundamental causes of disparities.
